# Metastatic Uterine Leiomyosarcoma as a Rare and Sinister Cause of Respiratory Distress: A Case Report and Literature Review

**DOI:** 10.7759/cureus.39101

**Published:** 2023-05-16

**Authors:** Laéshelle S Basanoo, Vishal Bahall, Salma Mohammed, Shravan Teelucksingh

**Affiliations:** 1 Intensive Care Unit / Anaesthetic Department, Sangre Grande Hospital, Sangre Grande, TTO; 2 Obstetrics and Gynaecology, The University of the West Indies, St Augustine, TTO; 3 Obstetrics and Gynaecology, San Fernando General Hospital, San Fernando, TTO; 4 Radiology, Sangre Grande Hospital, Sangre Grande, TTO

**Keywords:** uterine leiomyosarcoma, leiomyoma, pulmonary metastases, magnetic resonance imaging, immunohistochemistry, lactate dehydrogenase, caribbean, case report

## Abstract

Uterine leiomyosarcomas are an extremely rare subtype of uterine malignancy. This is a case report of a 47-year-old woman whose underlying uterine leiomyosarcoma manifested as acute respiratory distress secondary to pulmonary metastases. We highlight that a combination of suggestive imaging features and elevated lactate dehydrogenase (LDH) may prompt its diagnosis, notwithstanding that histological examination of a tissue sample is mandatory for its confirmation. The diagnosis of this condition is arduous for a multitude of reasons, including the insidious clinical course, aggressive nature, and high propensity to metastasize, coupled with a lack of standardised guidelines for its preoperative work-up. These challenges are amplified where resources may be limited, such as in the Caribbean region, where radiographic imaging and treatment options may not always be readily available.

## Introduction

Uterine leiomyosarcomas account for 2%-5% of all uterine malignancies [1], with an annual incidence of 0.35-0.64 per 100,000 women [2]. They convey a poor prognosis [3], and as such, distant metastases may be present in up to 81.4% of patients [4], with dissemination most commonly noted in the lung [1].

The diagnostic challenges encountered with leiomyosarcomas, especially in premenopausal women, are well established [1,3-12]. Owing to similarities in their clinical presentation, uterine leiomyosarcomas may be misdiagnosed as uterine leiomyomas [3,10-12], which are prevalent and present in up to 80% of women [13].

Features that increase one’s suspicion of leiomyosarcomas include age over 40, obesity, a single uterine mass, a rapidly enlarging mass, continued mass growth during menopause, prior history of tamoxifen or oral contraceptive pill use, menopausal use of oestrogen and/or progesterone, pelvic radiation, and the presence of ascites [3,9-12].

Currently, the most dependable pre-operative investigations for leiomyosarcomas are the combination of magnetic resonance imaging (MRI) and elevated lactate dehydrogenase (LDH) levels [11]. Despite MRI imaging advancements, there are still overlapping radiological features between leiomyomas and leiomyosarcomas that are onerous to distinguish. Additionally, MRI services are costly and not readily available in most public hospitals in the Caribbean [14].

This case report demonstrates a 47-year-old woman who presented with respiratory distress secondary to metastatic lung disease from an undiagnosed primary uterine leiomyosarcoma. This report aims to highlight the rare presentation and diagnostic challenges associated with this clinical entity in the Caribbean.

## Case presentation

**Figure 1:**
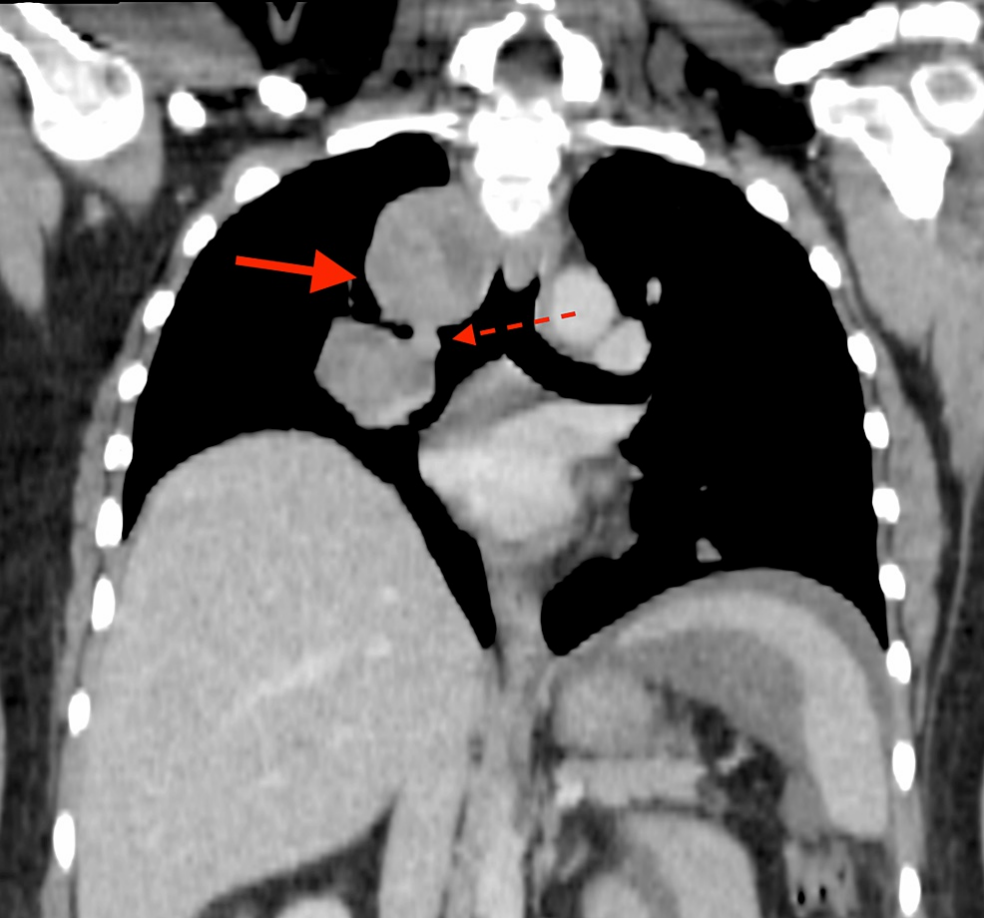
Coronal CT image post IV-contrast showing a large lobulated right hilar and paratracheal soft tissue mass (solid arrow) encasing the right main and upper lobe bronchus with partial occlusion of the latter (dashed arrow)

This is the case of a 47-year-old Indo-Caribbean woman with a body mass index (BMI) of 41 who presented to the emergency department with worsening dyspnea (exacerbated by a concomitant viral lower respiratory tract infection), pleuritic chest pain, and generalised weakness. On examination, she was also found to be tachycardic and diaphoretic. Computed tomography pulmonary angiography (CTPA) ruled out a potentially life-threatening pulmonary embolism but revealed a right-sided hilar mass (7.1cm AP x 5.1cm TS), encasing and partially occluding the right upper lobe bronchus (Figure 1).

**Figure 2:**
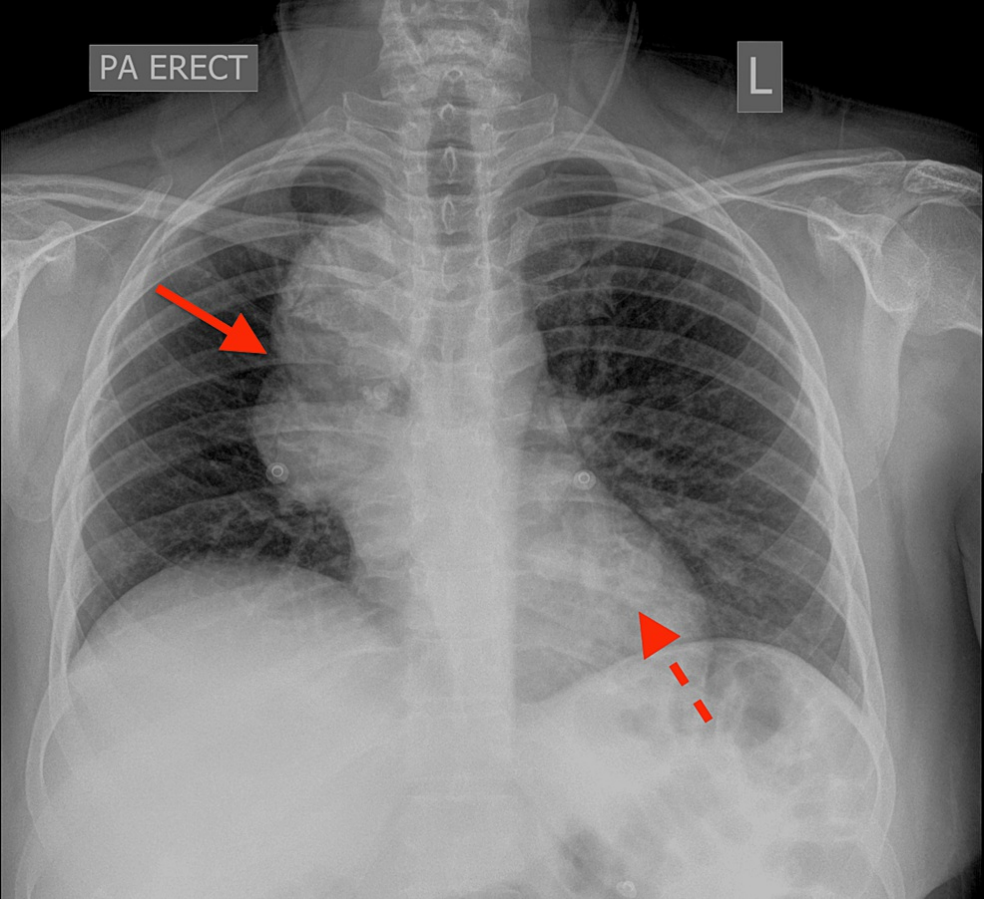
Postero-anterior chest X-ray demonstrating a large lobulated right perihilar and right paratracheal mass (solid arrow), as well as multiple contralateral left pulmonary nodules, the largest in the left retrocardiac region (dashed arrow)

It also demonstrated para-tracheal and para-aortic lymphadenopathy with pulmonary metastases dispersed bilaterally (Figure 2). The patient’s respiratory parameters were stabilised with medical management, and she was counselled on the need for further evaluation of her lung mass.

**Figure 3:**
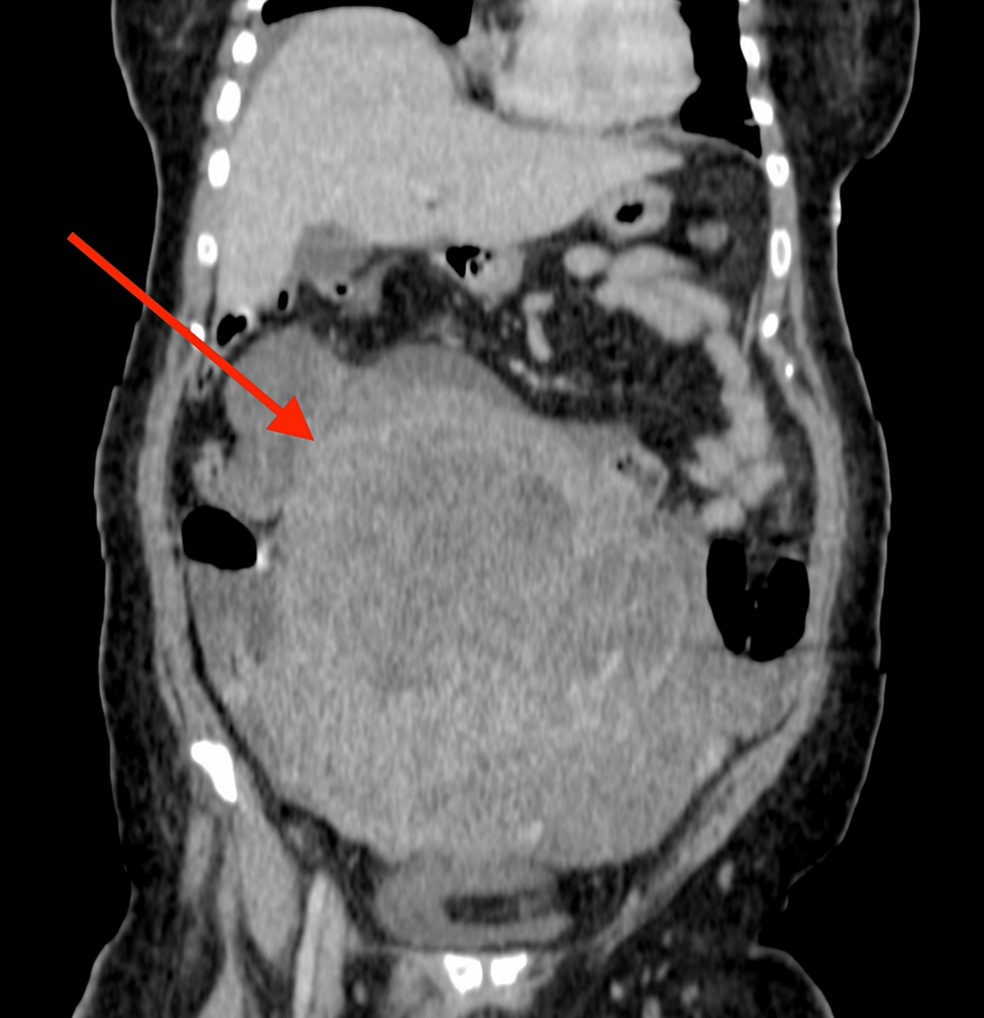
Coronal CT image of the abdomen post IV-contrast showing a large heterogeneously enhancing soft tissue mass arising from the pelvis (solid arrow) with ill-defined borders and surrounding free fluid

**Figure 4:**
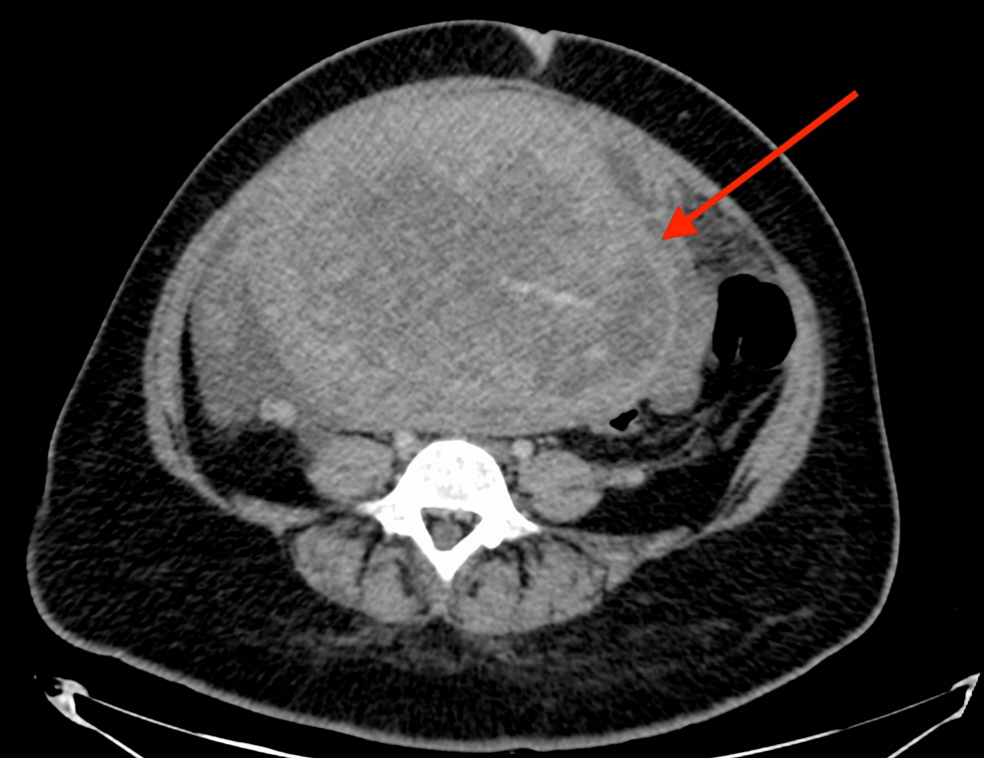
Axial CT image of the abdomen post IV-contrast scan showing a large heterogeneously enhancing uterine soft tissue mass (solid arrow) with internal neovascularization and surrounding free fluid

The patient subsequently defaulted on management but returned four months later with the aforementioned symptoms, in addition to weight loss and a pelvic mass. A full-body CT scan maintained similar chest findings; however, a large, lobulated, and heterogeneous uterine mass was discovered, likely a leiomyosarcoma (Figures 3, 4).

A multidisciplinary approach, involving gynecologic-oncology, medical oncology, radiology, and thoracic surgery, was undertaken to guide further diagnostic biopsies as her shortness of breath continued to worsen. A flexible bronchoscopy was performed, and a tissue sample was sent for further evaluation. Histology revealed spindled cells with increased mitoses, inclusive of abnormal forms with > 10/10 high power fields, consistent with a spindle cell neoplasm. Immunohistochemistry was supportive of a metastatic uterine leiomyosarcoma with strongly positive alpha smooth muscle actin.

The patient succumbed to respiratory failure days after bronchoscopy, prior to obtaining a sample of the uterine mass.

## Discussion

Uterine leiomyosarcomas are a rare type of malignant neoplasm that originate de novo from uterine smooth muscle cells, with only 0.2% due to sarcomatous transformation of a leiomyoma [9]. Risk factors for the development of this cancer include age over 40 and obesity, as observed in this patient. Hormonal therapies (tamoxifen, exogenous oestrogen, and/or progesterone use), African ancestry, exposure to pelvic radiation, and rare genetic associations such as retinoblastomas and Li-Fraumeni syndrome are also recognised [1,3, 8-11,15]. Traditionally, patients present with abnormal vaginal bleeding, a palpable pelvic mass, pelvic pain, and compressive symptoms resembling those of uterine leiomyomas [3,9,10,12,15] compounding its diagnostic challenges. Features more in keeping with leiomyosarcomas include accelerated growth or continued growth into menopause, whereas leiomyomas are generally slow growing and characteristically shrink during menopause. Additionally, leiomyosarcomas are a singular mass, whereas leiomyomas are frequently multiple masses dispersed throughout the uterus. Its aggressive metastatic pattern also infers that patients may present with complications of spread [9], such as acute respiratory distress secondary to pulmonary metastases, as demonstrated in our patient. Other features of metastases, such as ascites, pelvic lymphadenopathy, and constitutional symptoms, namely weight loss, generalised weakness, and fatigue, may be noted in leiomyosarcomas but not in leiomyomas, as the latter are benign with negligible metastatic potential [3,9,10,12,15].

While there is a wide array of investigative modalities, the preoperative diagnosis of uterine leiomyosarcomas continues to be a challenge [1,3-12]. This is further compounded by a paucity of evidence to develop specific preoperative diagnostic guidelines [3,9,11,12] and limited resources within the Caribbean healthcare setting [14]. Abdomino-pelvic ultrasound imaging is the first line of investigation for uterine pathology [7,12]. Although it is readily available and inexpensive, it is user-dependent and has limited penetrative depth, which restricts its accuracy [12]. Magnetic resonance imaging (MRI) is the preferred radiographic modality for distinguishing between leiomyomas and leiomyosarcomas, as it allows greater differentiation of soft tissue types [15]. The standard radiographic characteristics of leiomyomas include solitary or multiple distinct, spherical masses with well-delineated margins [3,7,9,12]. Comparatively, leiomyosarcomas are usually a singular mass with ill-defined borders, infiltrative growth patterns, and areas of hemorrhagic necrosis [3,7,9,12]. Blurred lines exist with atypical leiomyomas, leiomyomas that have undergone red degeneration, or those that contain fat (lipoleiomyomas), as they now mimic similar MRI findings to leiomyosarcomas [3,7,9,12,15]. An association also exists with uterine masses larger than 8 cm being more aligned with leiomyosarcomas as opposed to leiomyomas [3]. Computed tomography (CT) and positron emission tomography (PET) scans are not indicated in the differentiation between leiomyomas and leiomyosarcomas but are a reliable staple in the staging of patients with the latter [8,12,15].

A combination of supportive MRI features, including degenerative changes, along with elevated serum LDH levels are the most reliable preoperative modalities suggestive of uterine sarcomas [3,11]. The use of cancer antigen (CA) -125 has been evaluated; however, there is insufficient evidence to support it as a biomarker despite its being elevated in numerous cases [3,11,16], including this case (CA-125: 259 U/mL (range: 0-35 U/mL)). It is noted that our patient had a large uterine mass with dimensions of 14cm x 16cm x 17 cm and an elevated LDH of 915 U/L (range: 135-225 U/L), suggesting the diagnosis of leiomyosarcoma. Histological confirmation is needed for a definitive diagnosis and is usually established post-operatively [3,6,7]. This patient's diagnosis was confirmed upon immunohistochemical evaluation of the lung mass, which demonstrated positive oestrogen and progesterone receptors, representing metastases of uterine origin. Moreover, the sample exhibited strongly positive alpha smooth muscle actin, which is the most sensitive parameter for leiomyosarcomas, on immunohistochemical examination [5].

Delayed or inaccurate diagnosis and treatment of uterine leiomyosarcomas result in tumour progression and subsequent metastasis [3], as they are strongly associated with a high propensity for hematogenous spread [1,6,8]. Surgical intervention is the treatment of choice, with an en bloc total hysterectomy without morcellation [1,8,10,17]. Care should be taken to prevent intraoperative rupture and spillage of the mass, aiming to minimise dissemination. For advanced disease, the first-line chemotherapeutic agents are doxorubicin-based or gemcitabine combined with docetaxel, with newer regimes under consideration [8,15,17,18]. There is a wide variety of metastatic sites for uterine leiomyosarcomas, but the most common are the lung (74%), peritoneum (41%), bone (33%), and liver (27%) [1,4]. Distant metastases are a key prognostic factor; as documented in a review, all patients with metastatic uterine leiomyosarcoma died within five years [8]. The five-year disease-free states for stages I to IV are 75.8%, 60.1%, 44.9%, and 28.7%, respectively [1,17].

Given the nature of uterine leiomyosarcomas, there are obstacles encountered in their diagnosis and treatment. These challenges are further magnified in developing countries, where resources are limited. This case highlights the unusual presentation of acute respiratory distress from a metastatic uterine leiomyosarcoma and the importance of a multidisciplinary team approach in the management of these complex cases. It also explores ways in which radiological and haematological investigations can be combined to optimise and expedite diagnosis and, subsequently, treatment. We hope that this case report adds to the existing literature as well as contributes to research ventures that may develop diagnostic and treatment algorithms for uterine leiomyosarcomas to improve patient outcomes.

## Conclusions

Uterine leiomyosarcomas are a rare subtype of uterine malignancy. The unusual presentation of respiratory distress secondary to lung metastases in this patient contributed to the established diagnostic challenges. A high index of suspicion, along with the appropriate radiological and haematological investigations, are required for the earlier diagnosis and treatment of leiomyosarcomas. We hope that this case report adds to the existing literature and evidence on this clinical entity so that improvements in patient management and overall outcomes can be achieved.
